# Case report: Endoscopic closure with double stenting and autologous fascia lata graft of large tracheo-esophageal fistula

**DOI:** 10.3389/fsurg.2023.1107461

**Published:** 2023-04-27

**Authors:** Francesco Mattioli, Edoardo Serafini, Alessandro Andreani, Gaia Cappiello, Daniele Marchioni, Massimo Pinelli, Roberto Tonelli, Enrico Clini, Alessandro Marchioni

**Affiliations:** ^1^ Department of Otolaryngology Head and Neck Surgery, University Hospital of Modena, Modena, Italy; ^2^ Respiratory Diseases Unit, Department of Medical and Surgical Sciences, University Hospital of Modena, University of Modena Reggio Emilia, Modena, Italy; ^3^ Division of Plastic Surgery, Department of Medical and Surgical Sciences, University of Modena and Reggio Emilia, Modena, Italy

**Keywords:** tracheoesophageal fistula (TEF), fascia lata autograft, tracheoscopy, bronchoscope, stent

## Abstract

**Introduction:**

Radiotherapy and esophageal stenting are usually employed to manage esophageal localization of distant cancer. However, they are also related to the occurrence of an increased risk of tracheoesophageal fistula. Tracheoesophageal fistula management in these patients involves dealing with poor general conditions and short-term prognosis. This paper presents the first case in literature of bronchoscopic fistula closure through an autologous fascia lata graft placement between two stents.

**Case report and aim:**

A 67-years-old male patient was diagnosed with pulmonary squamous cell carcinoma in the inferior lobe of the left lung with mediastinal lymph node metastasis. After a multidisciplinary discussion, bronchoscopic repair of tracheoesophageal fistula with autologous fascia lata was decided without the removal of the esophageal stent due to the high risk on the esophagus possibly related to such a procedure. Oral feeding was progressively introduced without the development of aspiration symptoms. Videofluoroscopy and esophagogastroduodenoscopy were performed at 7 months showing no signs of tracheoesophageal fistula patency.

**Conclusion:**

This technique might represent a low risks viable option for patients unsuitable for open surgical approaches.

## Introduction

Acquired tracheoesophageal fistula (TEF) management is a challenging condition, as life expectancy with supportive care alone is reported to be 1–6 weeks (1). Based on etiology, TEF can be divided into malignant and benign, with each type making up approximately half of the acquired cases (2).

Esophageal localization of primary or distant malignancies, with tumor invasion through the walls of esophagus and trachea, is the main cause of TEF. Esophageal malignancy usually requires local treatments including esophageal stent positioning and radiotherapy (RT) to relieve symptoms and reduce tumor infiltration. However, these local treatments are burdened by an increased risk of TEF development due to the persistent pressure to the walls of esophagus and airway after esophageal stenting, which results in mucosal ischemic damage, and to radiotherapy-induced tissue necrosis (3).

The main treatments of TEF include interventional technique (bronchoscopy and endoscopy) or surgery. However, surgical procedures, such as fistula closure with pedicled muscle flap or omentum major, are rarely performed given the severe impairment of general conditions and the poor outcome of these patients. Interventional technique is the most common approach through esophageal and/or airway stenting to seal the fistula and prevent leakage (4). Despite most patients with TEF are successfully managed through an esophageal stent alone, double stenting (esophageal and airway stenting) should be considered in case of large fistulas (>2 cm), or when esophageal stent expansion results in airway compression or laceration.

Nevertheless, double stenting might be associated with further ischemia of the remaining tracheal and esophageal wall, compromising the healing process and resulting in a progressive widening of the fistula over time (1). Based on these considerations, autologous graft positioning between the two stents could reduce the unfavorable effects deriving from excessive esophago-tracheal wall compression, and could promote tracheal laceration repair, allowing to restore tracheal wall integrity. However, several issues should be considered when choosing a biomaterial for implantation in order to obtain a successful tracheal and esophageal healing with good clinical outcome. These include the type of immune response, the feasibility of the graft, and the possibility of autograft revascularization.

Here we present the first case of bronchoscopic TEF management using autologous fascia lata graft for a patient that resulted not eligible for open surgical treatment.

## Case description

A 67-years-old male patient was diagnosed with locally advanced non-small cell lung cancer (NSCLC) (clinical oncological staging: T3 N3 M0) with subcarinal lymph node metastasis causing esophageal compression. [^18^F]Fluorodeoxyglucose (FDG)-positron emission tomography (PET) confirmed an increased metabolic activity on the lower left lobe, site of the primary neoplastic tissue, and in correspondence with the metastatic lymph nodes at the level of the esophageal stenosis [standardized uptake value (SUV) max 10.2]. Considering the dysphagia reported by the patient, esophagogastroduodenoscopy (EGDS) was performed showing a 6 cm long intrinsic neoplastic stenosis in the caudal part of the esophagus. A fully covered metal esophageal stent was then positioned, leading to dysphagia resolution. After a multidisciplinary discussion with oncologist and radiotherapist, concomitant radiotherapy and chemotherapy (cCRT) was started. After the treatment, a restaging with computer tomography (CT)-scan and FDG-PET was performed, and a partial response (PR) was defined based on Response Evaluation Criteria in Solid Tumor (RECIST).

After a 17-months follow-up without disease progression, the patient developed cough, chest pain and pneumonia. The patient underwent chest CT-scan, which detected the development of TEF and inhaled pneumonia.

**Figure 1:**
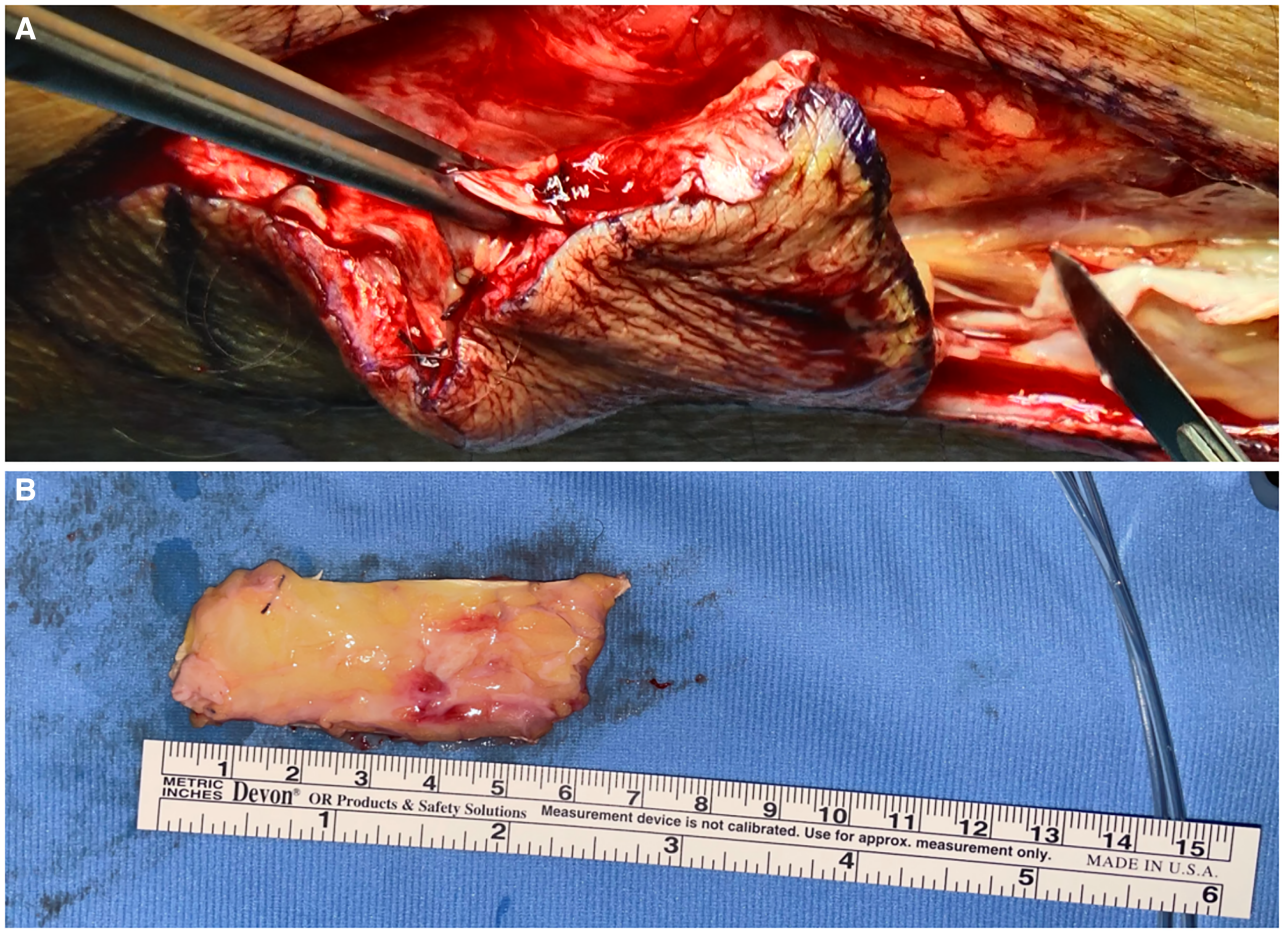
(**A**) Fascia lata harvesting with skin and subcutaneous tissue; (**B**) fascia lata graft after skin removal with subcutaneous fat tissue.

Urgent bronchoscopy was performed showing a roughly 3 cm × 1 cm TEF superior to the carina with the esophageal stent protruding into the tracheal lumen (Figure 1). A multidisciplinary discussion was made including bronchoscopists, otolaryngologists, plastic surgeons and anesthesiologists. Surgical correction was excluded due to high operative risk. However, given the patient's life expectancy after cCRT and the absence of disease progression after 17 months from treatments, an alternative procedure was proposed to allow the reconstruction of the tracheal laceration avoiding the progression of TEF secondary to a double stenting. The multidisciplinary team expressed consensus over a bronchoscopic repair of TEF with autologous fascia lata was decided, but without the removal of the esophageal stent due to the high risks on the esophagus such a procedure would have involved.

### Surgical technique

General anesthesia was induced and spontaneous breathing mantained. A Dumon rigid bronchoscope was employed (Efer Medical, La Ciotat, Cedex, France). A 5 cm long incision was performed along the lateral aspect of the caudal part of the thigh and a fascia lata graft of roughly 7 cm × 3 cm and 6 mm in thickness with subcutaneous fat tissue was harvested to obtain sufficient graft thickness (Figure 1).

Under bronchoscopic guidance TEF was identified and margins were freshened using a suction instrument. Synthetic biodegradable cyanoacrylate glue (Glubran 2;GEM) was injected on the edges of the tracheal laceration using a syringe *via* 5 Fr angioplasty catheter [Angiographic Catheter Tempo ® Vertebral (VERT) Ref. 451-514H0 Cordis ®] to create an elastic film on the posterior wall of the trachea and facilitate graft adhesion to the surrounding tissue. The fascia lata graft was taken with endoscopic forcep and positioned in the tracheal lumen passing through the rigid bronchoscope. The whole defect was fully covered with subcutaneous fat facing the esophageal stent and fascial side facing the tracheal lumen.

During graft placement, spontaneous breathing was suppressed in order to avoid fascia lata displacement.

**Figure 2:**
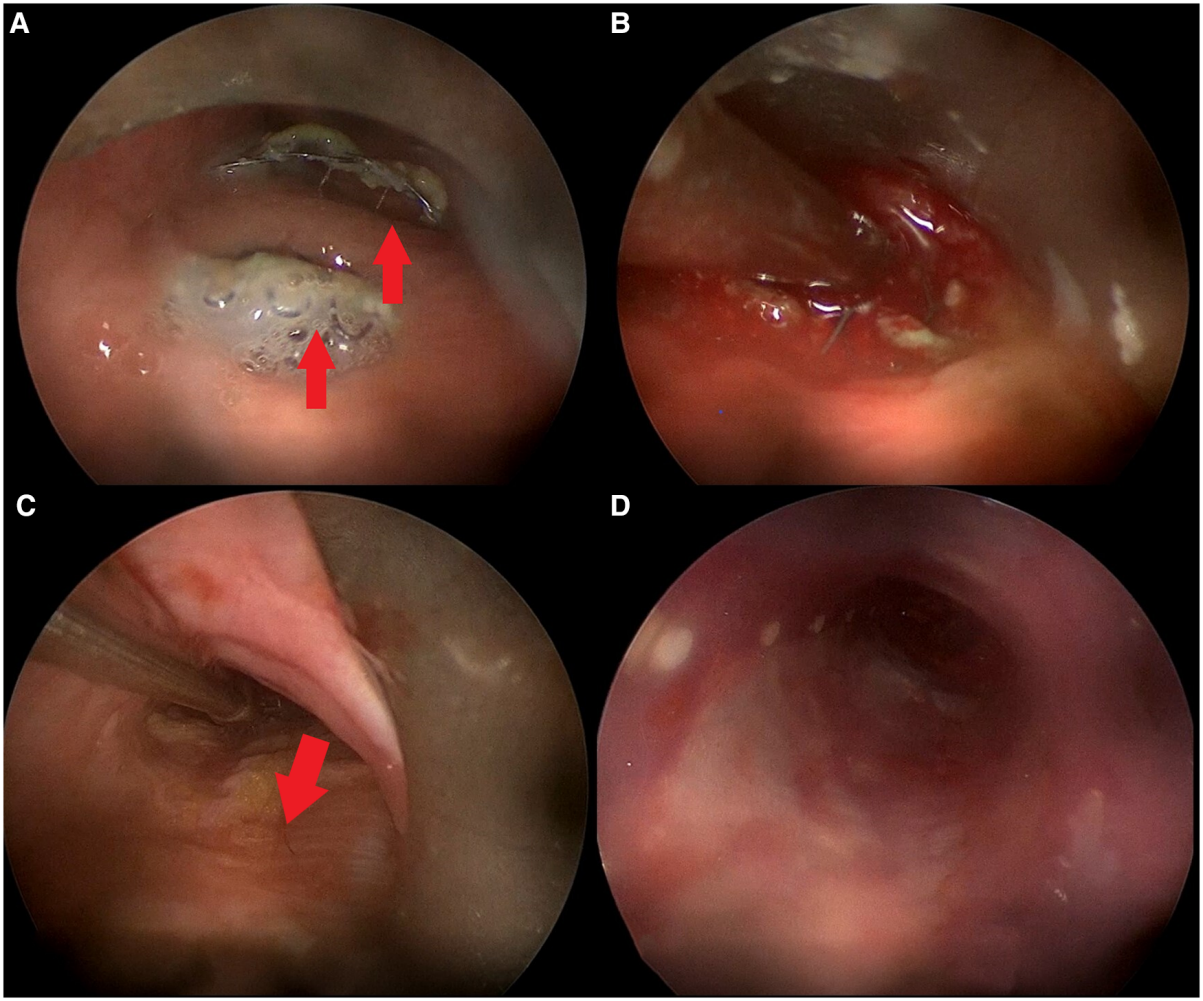
Intraoperative bronchoscopic procedure. (**A**) Rigid bronchoscopy showing TEF with esophageal stent protruding inside tracheal lumen in two different sites (red arrows); (**B**) TEF margins freshened using aspirator; (**C**) positioning of fascia lata (red arrow) over TEF with forceps; (**D**) tracheal stent correctly positioned over fascia lata graft.

A silicone tracheal stent (NOVATECH Dumon stents, ST 18-16-18 L 15-20-15, Boston Medical Products, Inc., Westborough, MA, USA) was placed to maintain the fascia lata graft in place allowing to cover TEF site both cranially and caudally (Figure 2). Flexible bronchoscopy was performed at the end of surgery in order to check the adequate graft placement.

**Figure 3:**
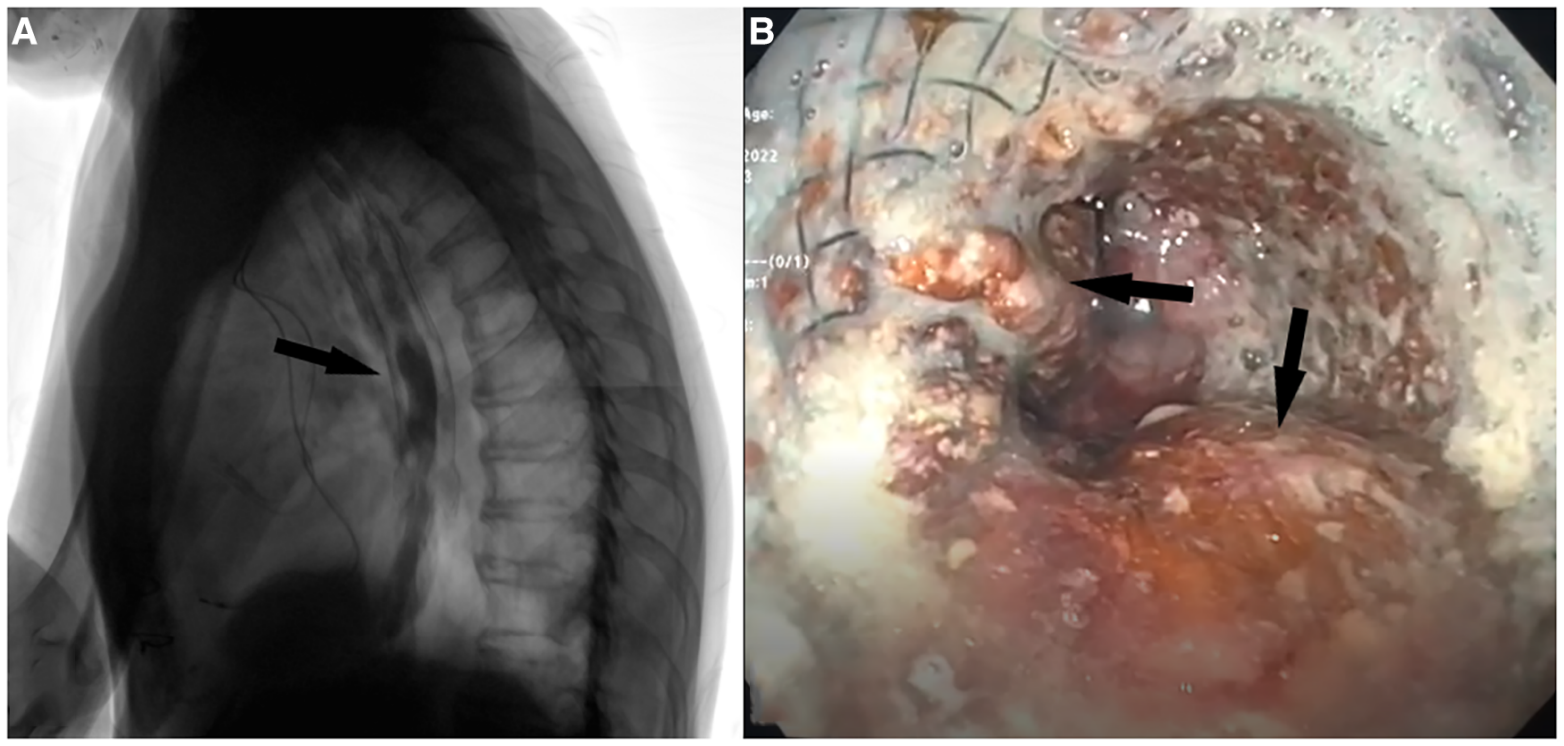
(**A**) Postoperative barium videofluoroscopy showing contrast medium passing regularly passing through the esophagus without signs of aspiration through the site of TEF. (**B**) EGDS performed after 7 months showing esophageal stent in place with fascia lata adipose tissue inside interstices (black arrows).

Videofluoroscopy was performed 1 week after the procedure showing no signs of TEF patency (Figure 3).

**Figure 4:**
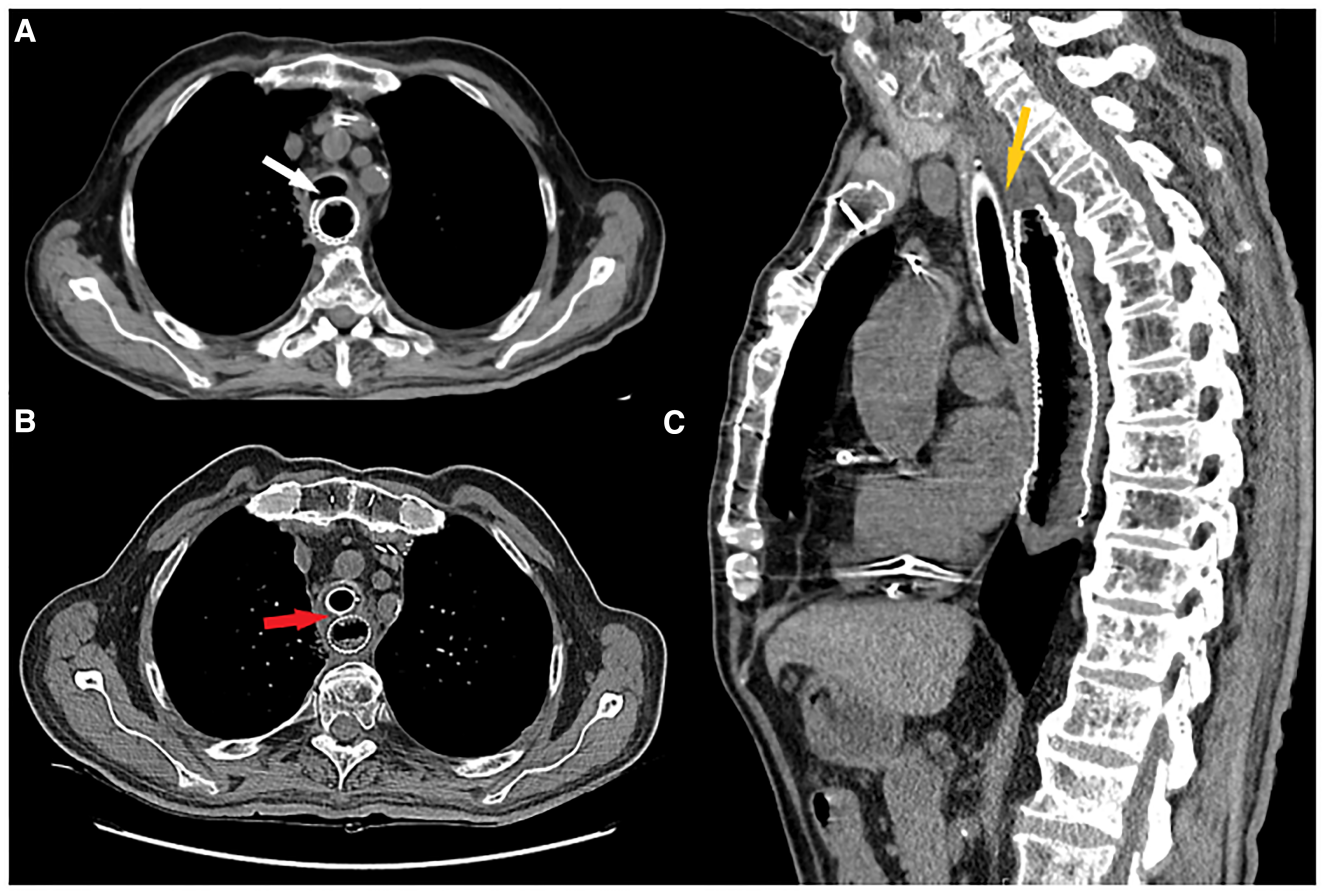
(**A**) Preoperative axial CT scan showing esophageal stent protruding inside tracheal lumen (white arrow). (**B**) Postoperative axial CT scan showing both esophageal and tracheal stent with fascia lata graft between them (red arrow). (**C**) Postoperative sagittal CT scan showing fascia lata graft (low density tissue between the two stents) interposed between the two stents covering the whole length of TEF.

Oral feeding was progressively introduced after 2 days. No aspiration symptoms were referred.

Videofluoroscopy and EGDS were performed after 7 months showing no signs of TEF patency. EGDS showed esophageal stent and fascia lata in place (Figure 3) with the patient maintaining oral feeding without any weight loss or aspiration symptoms or signs.

## Discussion

Acquired TEF is a challenging disease associated with very poor outcome. The goal of treatment is to prevent aspiration and to improve nutritional status. The choice of surgical approach depends on location and size of the fistula, and on the patient's clinical condition. However, given that surgery is often not feasible and is burdened with high complications and mortality, the endoscopic technique through stent placement is often preferred with the goal of covering the fistulous communication. Studies focusing on malignant TEF showed that airway and/or esophageal stent insertion provide an effective approach to improve quality of life (QoL), with a success rate for TEF closure ranging from 87% to 91% (5).

The introduction of esophageal self-expandable metallic stents (SEMS) allows to cover large defects with low rate of migration, maintaining esophageal patency, thus permitting enteral feeding and reducing bronchial contamination. Esophageal stenting using SEMS is one of the main procedures applied for palliative purpose in patients suffering from esophageal stricture due to cancer invasion. A major complication associated with the placement of esophageal SEMS includes pressure necrosis of the posterior tracheal wall with consequent TEF development. In one retrospective case-control study on 397 patients undergoing esophageal SEMS placement, 20 patients developed TEF after a median of 5 months following the procedure. However, despite TEF is an uncommon complication associated with esophageal stenting, patients suffering from multiple comorbidities treated with radiotherapy are at greater risk of tracheal wall fissurization (6).

In these cases, surgical procedures intended to repair TEF have to face with the difficulties of closing a fistula whose tissue margins are necrotic or even neoplastic, often on a patient exhibiting poor general conditions usually making him unsuitable for open surgical approaches.

Being esophageal stent removal before fistula repair not usually viable due to the high risk of further damage on the esophageal mucosa related to the procedure (7), double esophago-tracheal stenting was proposed among the main solutions. However, this treatment is usually associated with low clinical success (1) or progressive fistula widening due to further ischemic damage on the posterior tracheal wall and the anterior esophageal mucosa in the stenting site (8). Therefore, in patients with favorable response to oncologic treatments and with good performance status and life expectancy, the pressure from two stents on both sides of the tracheal membranous wall should be avoided to prevent TEF progression and healing process impairment. Combined autologous graft with double stenting could be an option to avoid contact between the two stents and to promote reconstruction of the posterior tracheal wall. Fascia lata is a favorable graft for its easy harvesting capability, mechanical proprieties and peculiar metabolic activity. Indeed, the characteristic flexibility of fascia lata could allow higher adaptability of the autologous graft to the physiological behavior of the tracheal wall during the act of breathing (9).

Fascia lata consists of a thick band of connective tissue with blood vessels, nerve and elastic fibers, allowing the adaptation of the fascia to the contraction of the muscle. Indeed, the “*crimped*” configuration of collagen fibers makes the fascia able to adapt to elongation, while the different orientation between layers allows the fascia to resist tensile stresses along various directions. Therefore, fascia lata graft could act as an excellent reconstructive material with mechanical proprieties allowing to maintain tracheal dynamic behavior during breathing. Furthermore, due to its relatively cellular nature and low nutritional requirements, tissue reaction or rejection is very limited.

The use of a fascia lata graft for TEF repair through a transcervical approach has been reported by some authors (10–12). Nevertheless, these reports focused on benign TEF and their primary outcome was to obtain a long-term closure. Dwivedi et al. described the management of an enlarged tracheoesophageal puncture through fascia lata autograft closure in two cases. However, this technique failed in one patient suffering from a larger fistula (12 mm diameter) with local infection and post radiotherapy changes (12).

Sugiyama A et al. described the successful use of a combined free autologous auricular cartilage and free fascia lata graft to repair a recurrent TEF after surgery for congenital esophageal atresia (11). Yalcinozan et al. reported a case of post-intubation TEF in which a fascia lata graft was successfully used as a reinforcement material for the esophageal anastomosis. At 12-months follow-up, the patient showed no food aspiration or dyspnea, confirming the effectiveness of the technique (10). The attempt of an endoscopic repair of TEF using autologous fascia lata had not been described.

The difficulty of the endoscopic intervention concerns keeping the graft in place and maintaining subsequent viability of the fascia lata through neovascularization from neighboring tissues. However, the technique we performed, with the use of biological glue and silicone stent, proved to be effective in keeping the graft in place, and the 7-month endoscopic control showed the presence of trophic fascia lata on the anterior wall of the esophagus where the TEF was previously located.

Stent selection is crucial for the success of endoscopic treatment. We used a silicone stent (NOVATECH Dumon stents, Boston Medical Products, Inc., Westborough, MA, USA; GSS™TD OD 18 mm, L 50 mm) with a diameter large enough to keep the underlying fascia compressed between the two stents, with the adipose layer facing the tracheal laceration (Figure 4).

The procedure described in our paper is aimed at avoiding TEF pulmonary complications, allowing at the same time oral feeding with the lowest risk of procedure-related complications in a patient deemed ineligible for open surgery. Still, this is a single case report, as such further patient sample will be necessary in order to validate this new technique.

## Conclusion

This is the first description of endoscopic TEF repair through fascia lata graft placement between the two stents, therefore further experience is still required to confirmed if this technique might overcome the double stent related complications due to direct contact between the two stents in patients with a favorable life expectancy. However, in patients who are not eligible to open transcervical or transthoracic approaches, the described technique might represent a viable option burdened with low morbidity and mortality risks related to the procedure.

## Data Availability

The raw data supporting the conclusions of this article will be made available by the authors, without undue reservation.
